# Measurement of Cerenkov Radiation Induced by the Gamma-Rays of Co-60 Therapy Units Using Wavelength Shifting Fiber

**DOI:** 10.3390/s140407013

**Published:** 2014-04-21

**Authors:** Kyoung Won Jang, Sang Hun Shin, Seon Geun Kim, Jae Seok Kim, Wook Jae Yoo, Young Hoon Ji, Bongsoo Lee

**Affiliations:** 1 School of Biomedical Engineering, College of Biomedical & Health Science, Research Institute of Biomedical Engineering, Konkuk University, 268 Chungwon-daero, Chungju-si, Chungcheongbuk-do 380-701, Korea; E-Mails: kko988@hotmail.com (K.W.J.); shshin9431@gmail.com (S.H.S.); chokomilkys@gmail.com (S.G.K.); kimzgo@naver.com (J.S.K.); wonzip@naver.com (W.J.Y.); 2 Research Center for Radiotherapy, Korea Institute of Radiological and Medical Sciences, 215-4, Gongneung-dong, Nowon-gu, Seoul 139-706, Korea; E-Mail: jyh328@kirams.re.kr

**Keywords:** Cerenkov radiation, wavelength shifting fiber, fiber-optic radiation sensor, radiation therapy

## Abstract

In this study, a wavelength shifting fiber that shifts ultra-violet and blue light to green light was employed as a sensor probe of a fiber-optic Cerenkov radiation sensor. In order to characterize Cerenkov radiation generated in the developed wavelength shifting fiber and a plastic optical fiber, spectra and intensities of Cerenkov radiation were measured with a spectrometer. The spectral peaks of light outputs from the wavelength shifting fiber and the plastic optical fiber were measured at wavelengths of 500 and 510 nm, respectively, and the intensity of transmitted light output of the wavelength shifting fiber was 22.2 times higher than that of the plastic optical fiber. Also, electron fluxes and total energy depositions of gamma-ray beams generated from a Co-60 therapy unit were calculated according to water depths using the Monte Carlo N-particle transport code. The relationship between the fluxes of electrons over the Cerenkov threshold energy and the energy depositions of gamma-ray beams from the Co-60 unit is a near-identity function. Finally, percentage depth doses for the gamma-ray beams were obtained using the fiber-optic Cerenkov radiation sensor, and the results were compared with those obtained by an ionization chamber. The average dose difference between the results of the fiber-optic Cerenkov radiation sensor and those of the ionization chamber was about 2.09%.

## 1. Introduction

Cerenkov radiation is produced in a dielectric material when a charged particle passes through the medium with a velocity greater than the phase velocity of light in the same medium [1]. This type of radiation can be easily observed in water at nuclear facilities such as boiling-water reactors, pressurized-water reactors, and spent fuel storage pools [2]. Here, burn-up of a fuel assembly can be estimated by measuring the intensity of Cerenkov radiation [3].

In radiotherapy dosimetry using fiber-optic radiation sensors (FORSs), however, Cerenkov radiation generated in plastic optical fibers (POFs) which are components of FORSs is frequently regarded as a severe noise signal. Since POFs are composed of dielectric materials, Cerenkov radiation can be generated in POFs by interacting with energetic charged particles. The previous development of radiotherapy dosimetry using FOSRs incorporates the measurement of light output from plastic and organic scintillator using a photo detector. Unfortunately, because the spectral range of Cerenkov radiation is very broad and covers that of scintillations from the organic scintillator, Cerenkov radiation generated in the POF is also acquired by the photo detector [4]. The most significant problem here is that the intensities of Cerenkov radiation generated in POFs vary as a function of the irradiated length of POFs. In various irradiation conditions such as varying field sizes and depths in a water phantom, absorbed doses obtained by a FORS without any correction for Cerenkov radiation generated in a POF are therefore different from those obtained by using only an organic scintillator. Several methods have thus been developed to remove or correct the Cerenkov radiation generated in a POF [5–7].

Cerenkov radiation signal from a POF can be significant and be used as a signal measurement that is related to the electron flux. In the case of therapeutic proton beams, Cerenkov radiation can be generated by subsequent electrons, and therefore Bragg peaks and spread-out Bragg peaks (SOBPs) of therapeutic proton beams can be obtained by measuring the intensities of Cerenkov radiation [8]. For therapeutic photon and gamma-ray beams, Cerenkov radiation is mainly produced by Compton electrons. Since Compton scattering is the predominant interaction for photon and gamma-ray beams, depth dose distribution depends on electron fluxes at each depth of a water phantom. Therefore, depth doses for therapeutic photon and gamma-ray beams can be obtained by measuring the intensities of Cerenkov radiation.

In previous works, relative depth doses for proton and photon beams were measured successfully using a fiber-optic Cerenkov radiation sensor (FOCRS) consisting of a pair of POFs. In radiotherapy dosimetry, the FOCRS has advantages such as a water-equivalent characteristic, non-quenching effect, and enhanced durability for therapeutic radiation [8,9]. However, since Cerenkov radiation generated in POFs is a supersubtle light signal, the sensor probe should be large enough (longer than 5 cm for a 1 mm-diameter POF) to produce a sufficient amount of Cerenkov photons to provide a reliable signal. In addition, although the spectral range of Cerenkov radiation is very broad, its intensities are mostly distributed in the ultra-violet (UV) and blue regions of the spectrum; in these regions, POFs have high attenuation coefficients [10] and therefore the intensities of Cerenkov radiation fade significantly.

To improve spatial resolution and Cerenkov collection efficiency of a FOCRS, a wavelength shifting fiber (WSF) that shifts UV and blue light to green light was employed in this study as a sensor probe of a FOCRS. By using a short length (in this research, 1 cm) of the WSF, it is possible to collect the reliable signals for Cerenkov radiation due to high UV to visible light conversion efficiency of the WSF. In order to characterize Cerenkov radiation generated in the WSF and a POF, spectra and intensities of Cerenkov radiation were measured with a spectrometer. Also, electron fluxes and total energy depositions of gamma-ray beams generated from a Co-60 therapy unit were calculated according to water depths using the Monte Carlo N-particle transport code (MCNPX). Finally, percentage depth doses (PDDs) for the gamma-ray beams were obtained using the FOCRS, and the results were compared with those obtained by an ionization chamber.

## 2. Materials and Methods

Throughout this study, a WSF (BCF-92, Saint-Gobain Ceramic & Plastics, Northborough, MA, USA) is employed to produce Cerenkov radiation. The WSF has a core/single-clad structure with 1 mm diameter and 1 cm length. A material density of the WSF is 1.05 g/cm^3^. The core of this fiber is synthesized with polystyrene (PS). The thickness of the polymethyl methacrylate (PMMA)-based claddings is approximately 4% of the fiber size. The refractive indices of the core and the cladding are 1.60 and 1.49, respectively, and the numerical aperture (NA) is 0.58. The NA denotes the light-gathering power, and more light can be guided by an optical fiber with a higher NA. The emission color and the peak wavelength of the WSF are green and 492 nm, respectively. In order to compare the gamma-ray induced light outputs of the WSF and a general optical fiber, a POF (BCF-98, Saint-Gobain Ceramic & Plastics) is also used to produce Cerenkov radiation. The components and geometrical dimensions of the POF are the same as those of the WSF.

**Figure 1.**
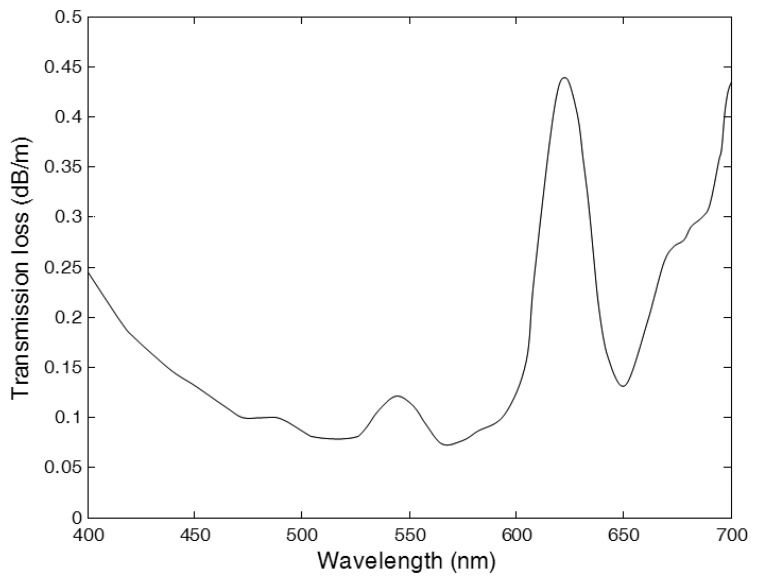
Transmission characteristic of the PMMA based optical fiber.

A commercial-grade multimode optical fiber (SH4001, Mitsubishi Rayon, Tokyo, Japan) is used to transmit light outputs generated from the WSF and the POF. The outer diameter of the fiber is 1.0 mm and the cladding thickness is 0.01 mm. Refractive indices of the core and the cladding are 1.49 and 1.40, respectively, and the NA is 0.51. The materials of the core and the cladding are PMMA and fluorinated polymer, respectively. The maximum transmission loss of the optical fiber is 0.09 dB/m for 500 nm collimated light. The transmission characteristic of the optical fiber can be found in Figure 1.

**Figure 2.**
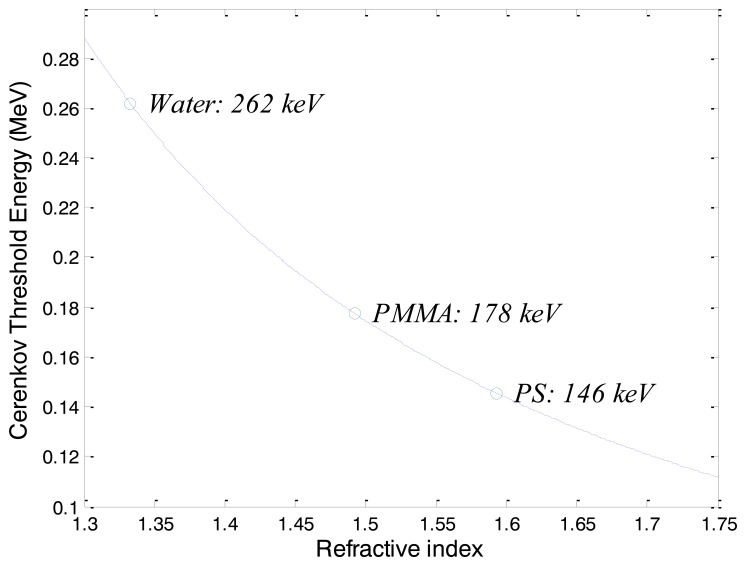
Cerenkov threshold energies of electrons according to refractive indices.

In general, charged particles should have sufficient energies to produce Cerenkov radiation in a dielectric material. Cerenkov threshold energies (CTE; E_Th_) of charged particles for the fibers (WSF, POF, and optical fiber for transmission) used in this study can be calculated using the special theory of relativity as follows [1]:

(1) $$E_{Th}=m_{0}c^{2}\left(\frac{n}{\sqrt{n^{2}-1}}-1\right)$$

where *m_0_* is the rest mass of a charged particle, *c* is the speed of light, and *n* is the refractive index. CTEs of electrons according to the refractive indices are shown in Figure 2. The CTEs of the electrons to produce Cerenkov radiation in the core materials of the fibers, including PMMA and PS, are 178 keV and 146 keV, respectively.

A spectrometer (AvaSpec-HS1024 × 122TEC, Avantes, Apeldoorn, The Netherlands) is used to measure the spectra and intensities of light outputs generated in the WSF and the POF. The measurable wavelength range of the spectrometer is between 200 and 1,160 nm. The signal-to-noise ratio (SNR) of the spectrometer is 60 dB.

The gamma-ray beams are provided by a Co-60 therapy unit (Theratron-780, AECL, Mississauga, ON, Canada). A Co-60 isotope has a half-life of 5.271 years and emits gamma-rays having energies of 1.173 and 1.332 MeV. The activity of the Co-60 isotope used in this study is about 3,000 Ci. The field size of the gamma-ray beams is 10 × 10 cm^2^ and the source to surface distance (SSD), which means the distance between the Co-60 isotope and the surface of the target, is 80 cm.

**Figure 3.**
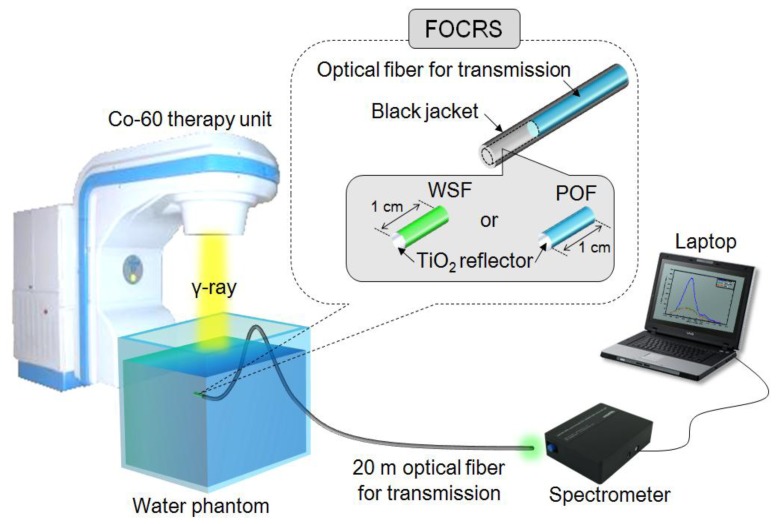
Structure of fiber-optic Cerenkov radiation sensors and experimental setup.

Figure 3 shows the structure of the FOCRSs and the experimental setup. When the gamma-ray beams are irradiated on the fibers, which are centered in the irradiation field, the light outputs are transmitted to the spectrometer through a 20 m-length optical fiber for transmission (SH4001). In order to measure the intensities of Cerenkov radiation according to the absorbed doses, light outputs from the fibers are acquired at various depths of a water phantom. In these experiments, Cerenkov radiation also is generated in the optical fiber for transmission, and therefore is removed by using the subtraction method. Typically, the subtraction method can be employed for measuring the difference between two sensor signals [11]. In our experiments, the intensities of Cerenkov radiation generated in the WSF and the POF are obtained by subtracting the signals of the transmission optical fiber from those of the FOCRSs. To increase the light collection efficiency, a TiO_2_ based reflective paint is coated at the end of the WSF and the POF.

**Figure 4.**
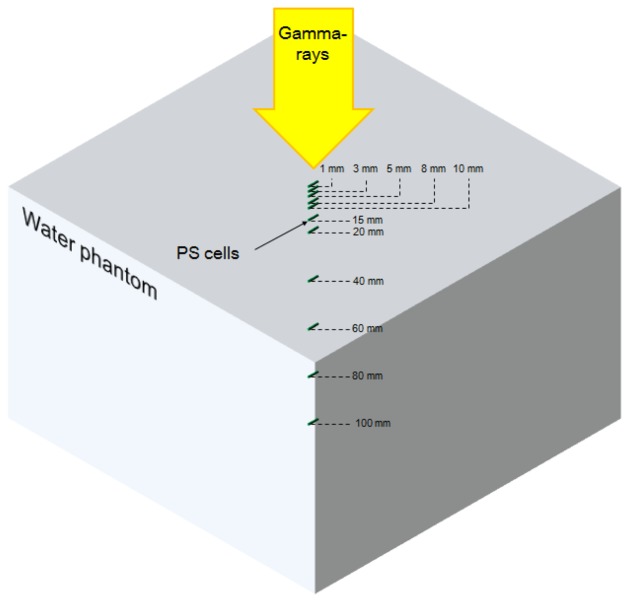
MCNPX scheme.

Using a MCNPX simulation, the fluxes of electrons over the CTE and energy depositions induced by the gamma-ray beams in the PS are calculated according to depths of a water phantom. In this simulation, a plane source produces gamma-rays having energies of 1.17 and 1.33 MeV. The field size of the gamma-ray beam is 10 × 10 cm^2^ and the SSD is 80 cm. The geometric dimensions of the water phantom are 50 × 50 × 50 cm^3^. PS cells having 1 mm-diameter and 1 cm-length are distributed as shown in Figure 4.

## 3. Results and Discussion

### 3.1. MCNPX Results

**Figure 5.**
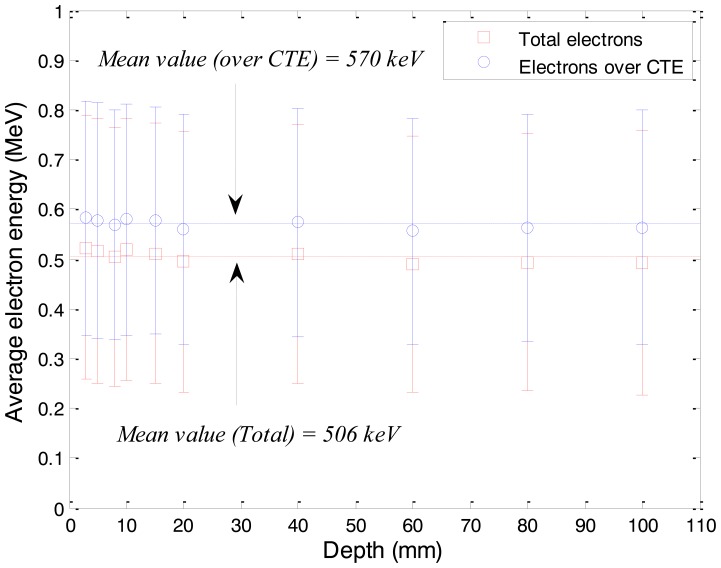
Average energies of electrons generated in polystyrene according to depths of a water phantom.

In order to produce Cerenkov radiation, energies of the subsequent electrons induced by the gamma-rays should be over the CTE. As mentioned above, the CTE of PS, the core material of the WSF, is about 146 keV. As shown in Figure 5, the average energy of total electrons induced by gamma-rays was calculated as approximately 506 keV, and this result reveals that the subsequent electrons induced by the gamma-rays have sufficient energies to produce Cerenkov radiation in the WSF. In the simulation result, we can also find the uniform distribution of electron energies according to water depths. This result shows that the fluctuations of relative depth doses for gamma-rays of the Co-60 are mainly caused by the electron fluxes, not the electron energies.

The average energies of the electrons over the CTE as a function of water depth also can be found in Figure 5. In general, the intensity of Cerenkov radiation (*I_C_*) generated by an electron per unit path can be obtained using the Frank-Tamm formula as [12]:

(2) $$I_{C}=2\pi \alpha z^{2}\frac{d\lambda }{\lambda^{2}}\left(1-\frac{1}{\beta^{2}n^{2}}\right)$$

where *α* is the fine structure constant (=1/137), *z* is the charge of the particle, *λ* is the wavelength of Cerenkov radiation, *n* is the refractive index of medium, and *β* is the velocity of particle relative to light. In cases of relativistic electrons, the value of *β* is close to “1” and the intensity of Cerenkov radiation is nearly independent from the energies of electrons [13]. In our simulation, the average energies of the electrons over the CTE according to water depths were maintained uniformly at approximately 570 keV. Therefore we can estimate that the intensities of Cerenkov radiation as a function of water depth are not strongly affected by electron energies.

**Figure 6.**
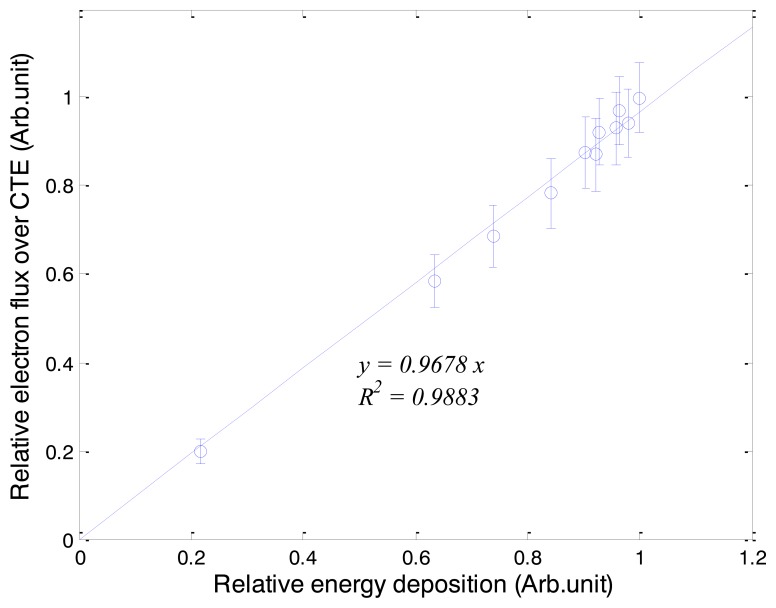
Relationship between fluxes of electrons over the CTE and energy depositions of the gamma-ray beams.

Figure 6 shows the relationship between fluxes of electrons over the CTE and energy depositions of gamma-rays. The results were obtained by one-to-one correspondence of the electron fluxes and the energy depositions according to water depths. In our simulation results, the relationship between the fluxes of electrons and the energy depositions is a near-identity function with a gradient of 0.97. This result shows that depth doses for gamma-ray beams are obtainable using the intensities of Cerenkov radiation.

### 3.2. Measurement of Cerenkov Radiation Using FOCRSs

**Figure 7.**
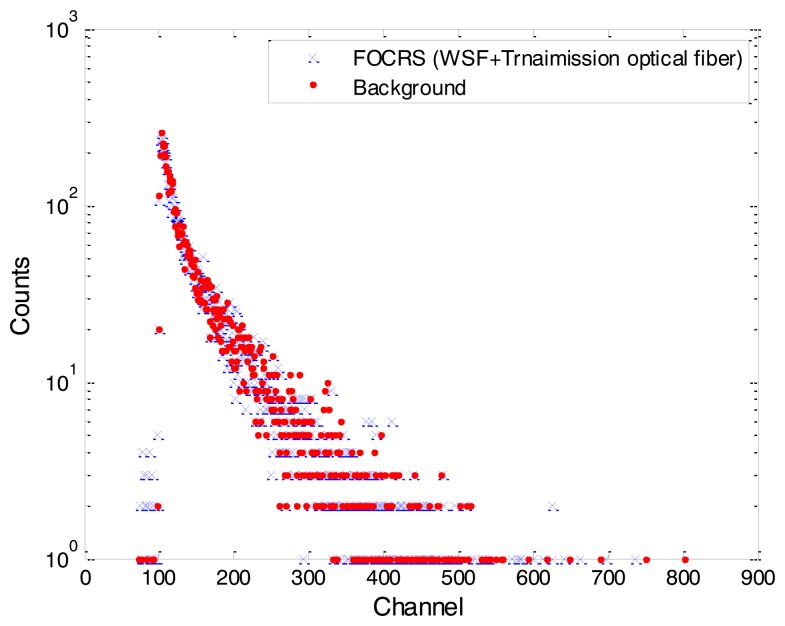
The response of the FOCRS incorporating the WSF for 88 keV gamma-rays emitted from a Cd-109.

First of all, since the WSF can produce light signals by interacting with low energy gamma-rays, we verified the response of the WSF for 88 keV gamma-rays emitted from a Cd-109 isotope. The response of the FOCRS incorporating the WSF for the gamma-rays of a Cd-109 with 1 μCi activity can be found in Figure 7. In this experiment, a photomultiplier tube (PMT; R11265-200, Hamamatsu Photonics, Hamamatsu, Japan)—amplifier (AMP; C7319, Hamamatsu Photonics)—multichannel analyzer (MCA; EASY-MCA-8k, ORTEC, Oak Ridge, TN, USA) system was exploited to measure the supersubtle light signals of the FOCRS. As a result, there was no significant difference between background signals and those of the FOCRS incorporating the WSF.

**Figure 8.**
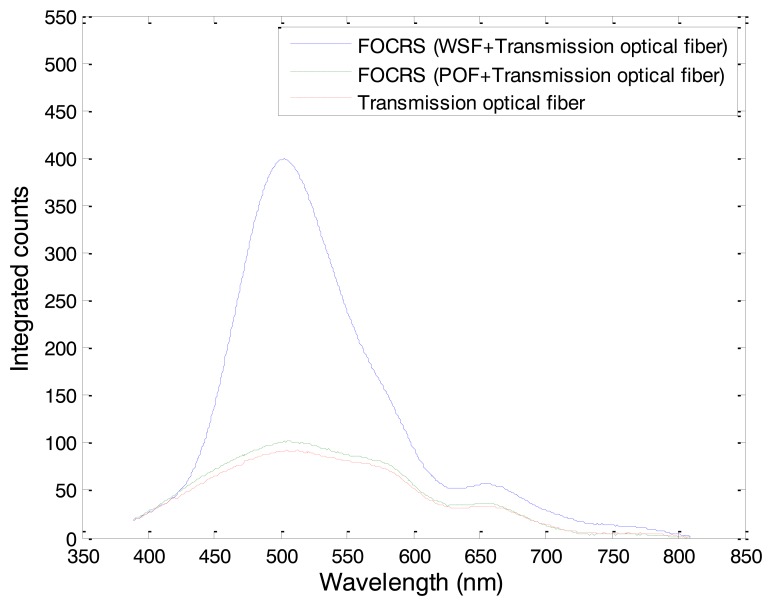
Spectra of light outputs generated in FOCRSs.

Figure 8 shows the spectra of light outputs generated in FOCRSs. The spectra were obtained by integrating the counts for 60 s of irradiation at 5 mm depth of a water phantom. In general, the intensity of Cerenkov radiation is proportional to a negative cube of the wavelength, and therefore the intensity of Cerenkov radiation increases toward shorter wavelengths [14]. In the case of the FOCRS including the POF and the optical fiber for transmission, however, the intensity of Cerenkov radiation has a peak at a wavelength of 510 nm. This discrepancy is caused by the attenuation characteristics of the optical fiber for transmission. In general, the PMMA based optical fibers have high transmission losses at short wavelengths and have attenuation peaks at wavelengths around 540 and 620 nm as shown in Figure 1. As a result, the measured spectrum of Cerenkov radiation has a similar trend as the characteristic transmission of the optical fiber. In this experiment, optical fiber for transmission with a length of 20 m was used, and thus the original wavelength of the Cerenkov radiation might be incorrectly obtained according to the transmission properties of the optical fiber. In the case of the FOCRS consisting of the WSF and the optical fiber for transmission, the peak was at a wavelength of 500 nm. As mentioned above, the WSF collects the light signals in the UV and blue regions and then converts them into measurable green signals. Therefore, the light output of the WSF was much higher than that of the POF when we subtract the intensity of Cerenkov radiation generated in the optical fiber for transmission from the signals of the FOCRSs. In this result, the light output of the WSF via the optical fiber for transmission was 22.2 times higher than that of the POF; here, the intensities were obtained by integrating the counts from 400 to 800 nm.

In general, the Cerenkov radiation has an emission angle (*θ*) which is determined by the refractive index (*n*) of the medium and the energy (*E*) of a charged particle as follows [15]:

(3) $$\theta =\cos^{-1}(\frac{1}{n\sqrt{1-\frac{1}{{\left(\frac{E}{m_{0}c^{2}}+1\right)}^{2}}}})$$

**Figure 9.**
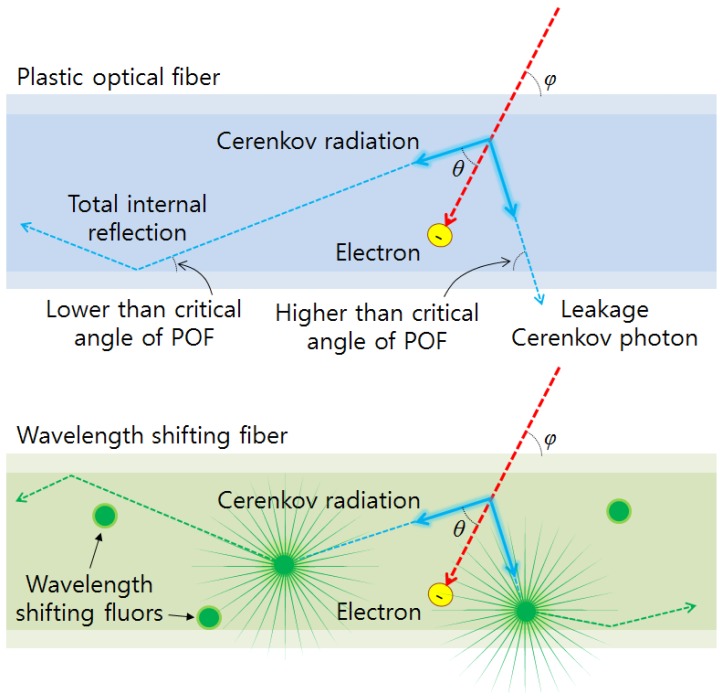
Transmission of light signals generated in a POF and a WSF.

In the previous simulation result, the mean energy of electrons over CTE produced in the PS based POF was about 570 keV. For this type of electron, the emission angle of Cerenkov radiation is about 44.8° in the PS (*n* = 1.6). Consequently, Cerenkov radiation generated in the POF has an angular dependence for the incident electron because the POF has a critical angle to transmit the light signals as shown in Figure 9.

**Figure 10.**
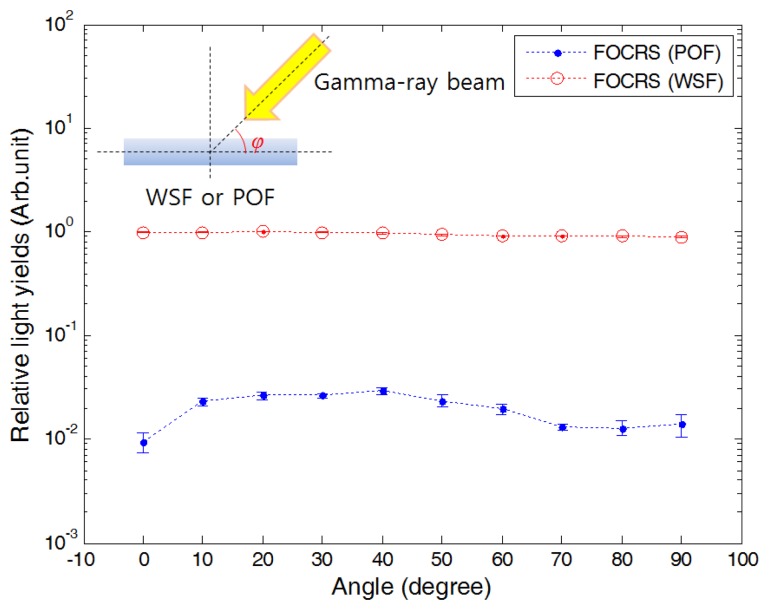
Angular dependence of FOCRSs.

Prior to measurement of PDDs, we should clarify the angular dependence of FOCRSs because scattering angles of electrons are not constant according to depths of a water phantom. The angular dependence of FOCRSs can be found in Figure 10. Here, the results were obtained by subtracting the light output of the optical fiber for transmission from those of FOCRSs. Typically, transmitted amount of Cerenkov radiation in a POF depends on the incident angle of electron and has a peak when the incident angle of electron is the same as the emission angle of Cerenkov radiation [16]. In our experiment, transmitted amount of Cerenkov radiation in the POF had a peak at the irradiation angle of 40°; this value is close to the theoretical Cerenkov emission angle for 570 keV electrons in the PS.

The angular dependence of the WSF can also be found in Figure 10. In the result, the light outputs generated from the WSF according to irradiation angles were maintained almost uniformly. The WSF contains wavelength shifting fluors. These kinds of fluors make it possible to reduce the angular dependence of the FOCRS and to increase the collection efficiency of Cerenkov radiation because they emit the converted photons with all directions as shown in Figure 9. Through this experiment, we can conclude that the response of WSF is almost independent from incident angles of electrons.

**Figure 11.**
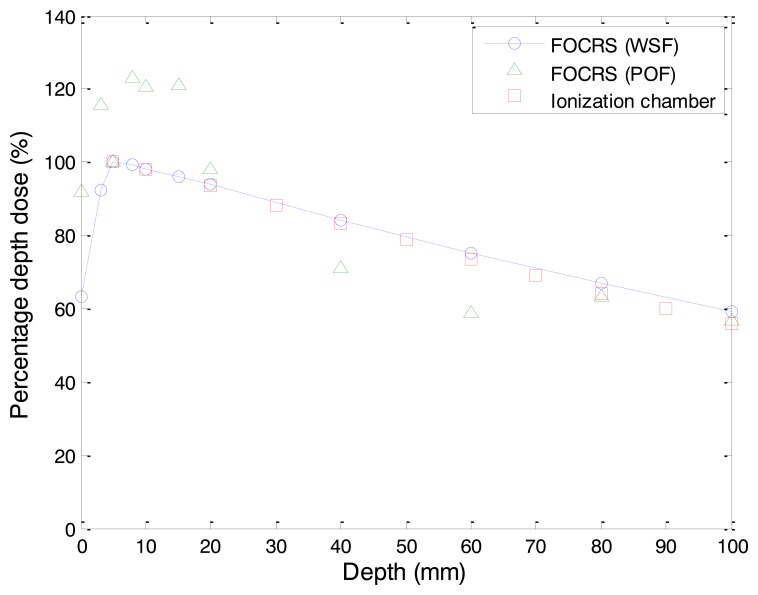
Measured percentage depth doses according to depths of water phantom using FOCRSs.

Measured PDDs in water can be found in Figure 11. In general, the central axis dose distribution is characterized by the PDD, which can be defined as the ratio of the absorbed dose at any depth (*d_t_*) to the peak absorbed dose at a fixed reference depth (*d_max_*) in the center of the beam fields. The PDD can be expressed as [17]:

(4) $$\text{PDD}(\%)=\frac{d_{t}}{d_{\max}}\times 100$$

The PDD is the ratio of dose at depth to dose at a fixed reference depth, expressed in percentage, and it varies according to SSD, energy, and field size. For gamma-ray beams emitted from the Co-60 therapy unit, PDDs increased steeply according to the depth of *d_max_*, and then decreased slowly. It has been observed that the typical depth of maximum dosage along the central axis for a 10 × 10 cm^2^ field in a water phantom is about 5.0 mm for gamma-rays emitted from the Co-60 therapy unit [17].

In our experiment, the depth of *d_max_* obtained by the FOCRS including the WSF was 5.0 mm. The PDDs obtained with a PTW Farmer ionization chamber are also plotted for comparison with those of FOCRSs. The ionization chamber exploited in this experiment has 0.6 cc sensitive volume with 6.1 mm diameter and 23.6 mm length. The wall material is graphite with a protective acrylic cover, and the electrode is made of Al. The average dose difference between the results of the FOCRS including the WSF and those of the ionization chamber was about 2.09%. In the case of the FOCRS including the POF, we could not obtain reliable data due to its small light output. From the results of this experiment, we can conclude that the WSF can be used effectively as a sensor probe of a FOCRS.

## 4. Conclusions

In this study, a FOCRS was fabricated using a WSF to measure Cerenkov radiation induced by gamma-ray beams of a Co-60 therapy unit. Based on MCNPX simulations, the relationship between fluxes of electrons over the CTE and energy depositions of gamma-ray beams of Co-60 could be determined. The relationship between the fluxes of electrons and the energy depositions is a near-identity function. To characterize Cerenkov radiation generated in the WSF and a POF, spectra and intensities of Cerenkov radiation were measured with a spectrometer. The spectral peaks of light outputs from the WSF and the POF were measured at wavelengths of 500 and 510 nm, respectively, and the intensity of transmitted light output of the WSF was 22.2 times higher than that of the POF. Finally, the PDDs for the gamma-ray beams of the Co-60 therapy unit were obtained using the FOCRS and the results were compared with those obtained from an ionization chamber. The average dose difference between the results of the FOCRS and those of the ionization chamber was about 2.09%.

Future studies will be carried out to fabricate a one-dimensional FOCRS for real-time and multi-dimensional measurements with multi-channel photo detectors such as a photodiode array and a position sensitive photomultiplier tube (PS-PMT).
